# Increasing and sustaining blood-borne virus screening in Spain and Portugal throughout the COVID-19 pandemic: a multi-center quality improvement intervention

**DOI:** 10.3389/fpubh.2023.1268888

**Published:** 2024-01-24

**Authors:** Inês Vaz-Pinto, Enrique Ortega, Ivan Chivite, María Butí, Juan Turnes-Vázquez, Vítor Magno-Pereira, Miguel Rocha, Jorge Garrido, Catarina Esteves-Santos, Mafalda Guimaraes, Tomás Mourão, María Martínez Roma, Vanessa Guilera, Jordi Llaneras-Artigues, Ana Barreira-Díaz, Santiago Pérez Cachafeiro, Sandra Daponte Angueira, Elisa Xavier, Mariana Vicente, Gema Garrido, Maria Teresa Heredia, Diogo Medina, Miguel García Deltoro

**Affiliations:** ^1^HIV-AIDS Functional Unit, Hospital de Cascais Dr. José de Almeida (HCASCAIS), Cascais, Portugal; ^2^Unidad de Enfermedades Infecciosas, Consorci Hospital General Universitari de València (HVALENCIA), Valencia, Spain; ^3^Unidad de Enfermedades Infecciosas, Hospital Clínic i Provincial de Barcelona (HClinic), Barcelona, Spain; ^4^Servicio de Hepatología, Hospital Universitari Vall d'Hebron (HVHEBRON), Barcelona, Spain; ^5^CIBER Enfermedades Hepáticas y Digestivas del Instituto Carlos III, Madrid, Spain; ^6^Servizo Galego de Saúde (SGALICIA), Galicia, Spain; ^7^Serviço de Saúde da Região Autónoma da Madeira (SMADEIRA), Madeira, Portugal; ^8^Universidade da Madeira, Madeira, Portugal; ^9^Grupo de Ativistas em Tratamentos (GAT), Lisbon, Portugal; ^10^Apoyo Positivo (A+), Torremolinos, Spain; ^11^Gilead Sciences, Madrid and Lisbon, Spain and Portugal

**Keywords:** blood borne viruses, screening, linkage to care (LTC), human immunodeficiency virus (HIV), HVB, HCV (hepatitis C), COVID-19

## Abstract

**Background:**

Around 57,000 people in Spain and Portugal currently living with HIV or chronic hepatitis C are unaware of their infection. The COVID-19 pandemic severely disrupted screening efforts for these infections. We designed an intervention to increase and sustain opportunistic blood-borne virus (BBV) screening and linkage to care (SLTC) by implementing the TEST model.

**Methods:**

The Plan Do Study Act (PDSA) method of quality improvement (QI) was implemented in 8 healthcare organizations (HCOs), including four hospitals, two clusters of community health centers, and two community-based organizations (CBOs). Baseline assessment included a review of BBV SLTC practices, testing volume, and results 12 months before the intervention. Changes in BBV testing rates over time were measured before, during, and after the COVID-19 lockdowns in 2020. A mixed ANOVA model was used to analyze the possible effect on testing volumes among HCOs over the three study periods.

**Intervention:**

BBV testing was integrated into normal clinical flow in all HCOs using existing clinical infrastructure and staff. Electronic health record (EHR) systems were modified whenever possible to streamline screening processes, implement systemic institutional policy changes, and promote QI.

**Results:**

Two years after the launch of the intervention in screening practices, testing volumes increased by 116%, with formal healthcare settings recording larger increases than CBOs. The start of the COVID-19 lockdowns was accompanied by a global 60% decrease in testing in all HCOs. Screening emergency department patients or using EHR systems to automate screening showed the highest resilience and lowest reduction in testing. HCOs recovered 77% of their testing volume once the lockdowns were lifted, with CBOs making the fullest recovery. Globally, enhanced screening techniques enabled HCOs to diagnose a total of 1,860 individuals over the research period.

**Conclusions:**

Implementation of the TEST model enabled HCOs to increase and sustain BBV screening, even during COVID-19 lockdowns. Although improvement in screening was noted in all HCOs, additional work is needed to develop strong patient linkage to care models in challenging times, such as global pandemics.

## 1 Introduction

Reaching and testing those at risk of infection with blood-borne viruses (BBV) such as human immunodeficiency virus (HIV), hepatitis B virus (HBV), or hepatitis C virus (HCV) remains a public health challenge in the Iberian Peninsula. An estimated 19,600 and 2,800 people living with HIV in Spain and Portugal, respectively, are unaware of their infection, and one in two receive their diagnosis at a late stage (CD4^+^ T cell count <350 cells/mm^3^ at diagnosis) (1–3). Similarly, an estimated 22,500 and 12,300 people living with chronic HCV in Spain and Portugal, respectively are unaware of their infection (4, 5). The prevalence of chronic HBV infection is estimated at 0.7% for both countries: 331,400 people live with chronic hepatitis B in Spain and 72,000 in Portugal, an unknown proportion of whom remain undiagnosed (6). Late BBV diagnosis is problematic not just for affected individuals, due to increased morbidity and mortality, but also for society, due to missed opportunities to break transmission (7).

Screening, a cornerstone of secondary prevention, is essential to reduce BBV prevalence and prevent further transmission (8). To date, BBV screening has typically followed traditional models, requiring dedicated staff and resources outside routine clinical practice (3). Instead of integrating screening into the regular provision of care for all eligible patients, traditional approaches often rely on a case-by-case decision, which may reinforce the stigma associated with testing for these infections.

National and international guidelines alike recommend enhancing BBV screening and linkage to care (SLTC) practices. In its evidence-based guidance on integrated BBV testing, the European Center for Disease Prevention and Control (ECDC) calls on countries to increase testing coverage and uptake in order to achieve the UN's epidemic control goals for 2030 (9–11). The Spanish Ministry of Health recommends HIV screening in sexually active individuals between the ages of 20 and 59 who present at primary care facilities, require a blood-draw for any clinical reason, and live in a Spanish province where HIV incidence in the previous 3 years has been above the 75th percentile (12). While it limits its recommendations on HCV screening to persons with a history of exposure to the virus or other known risk factors, the Spanish Ministry of Health recognizes that this strategy has not adequately addressed the undiagnosed population, and is currently undertaking a clinical efficacy and cost-effectiveness analysis of a birth cohort screening strategy for the Spanish population (13). The Portuguese General Directorate for Health, meanwhile, recommends screening patients aged 18 to 64 for HIV at least once in their lifetime, and progressively screening high prevalence population groups for HCV (14, 15).

Despite these recommendations, implementation of BBV SLTC in formal healthcare organizations (HCO) in Spain and Portugal is low and heterogeneous when compared to the role played by community-based organizations (CBO), relative to their size and resources (16).

In 2020, the advent of the SARS-CoV-2 pandemic led to decreased access to BBV prevention services and testing as health systems diverted resources toward fighting a global health crisis (17). Surveys of HIV specialists found that 53% to 58% were now treating patients with SARS-CoV-2 infection, as infectious disease specialists, internal medicine specialists and other healthcare personnel were needed to manage the pandemic (18, 19). In addition, 35% of survey respondents also reported disruptions in HIV testing services. Similar disruptions were noted for viral hepatitis screening (20). This disruption in screening programs and decelerated linkage to care could worsen BBV control (21, 22).

HCOs in different cities across the US have successfully used the TEST model to promote system changes and expand SLTC in various settings (23–27). TEST consists of 4 guiding pillars for enhanced SLTC: T, Testing and linkage integrated into the normal clinical flow, using existing clinical infrastructure and staff to create efficiencies; E, Electronic health record (EHR) modification, enhancing efficiencies within EHR and other technologies to facilitate appropriate screening; S, Systemic policy change, implementing institutional and regional policy change to support screening and linkage to care; and T, Training, feedback and continuous quality improvement. Training was performed on all organizations involving (1) a refresher on the basics of HIV and viral hepatitis infection and care, and (2) instructions on how to enroll patients in screening, including appropriate language for opt-out. Program data was utilized to track progress, to identify areas for improvement, and to support staff training (28). In other words, the aim of TEST is to take advantage of visits to medical facilities to offer eligible individuals on-the-spot testing for viruses while blood work is processed for other reasons. This opportunistic screening approach reduces patient biases while respecting their right to decline this or any other common clinical investigations (29).

The aim of this multi-center quality improvement intervention was to increase and sustain opportunistic BBV screening by implementing the TEST model. The project involved 8 HCOs, including 4 hospitals, 2 clusters of primary care centers, and 2 CBOs.

## 2 Materials and methods

### 2.1 Context

Eight organizations in Spain and Portugal participated in the study during different periods: Hospital de Cascais Dr. José de Almeida (further refered to as HCascais), in Cascais, Portugal; Consorci Hospital General Universitari de València (further refered to as HValencia), in Valencia, Spain; Hospital Clínic i Provincial de Barcelona (further refered to as Hclinic) in Barcelona, Spain; Hospital Universitari Vall d'Hebron (further refered to as HVHebron), in Barcelona, Spain; Servizo Galego de Saúde (further refered to as SGalicia), in Galicia, Spain; Serviço de Saúde da Região Autónoma da Madeira (further refered to as SMadeira), in Madeira, Portugal; Grupo de Ativistas em Tratamentos (further refered to as GAT), in Lisbon, Portugal and Apoyo Positivo (further refered to as A+), in Madrid and Malaga, Spain.

### 2.2 Eligibility criteria used in participating centers

HCascais: the intervention was implemented in the adult Emergency Department (ED) in September 2018. Criteria for HIV and HCV screening were: age 18 to 64 years; presenting to the ED; no record of previous serologies; need for phlebotomy for any reason.

HValencia: the intervention was implemented in 26 primary care sites of this Health department in València, in February 2019. Criteria for HIV, HBV, and HCV screening were: age 18 to 80 years; presenting to primary care; no record of previous serologies; need for phlebotomy for any reason.

HClinic: the intervention was implemented in the ED in January 2020. Criteria for HIV, HBV, and HCV screening were: age ≥16 years; presenting to the ED with genitourinary complaints or reporting recent high-risk exposures, such as chemsex, shared injecting materials, or unprotected penetrative intercourse.

HVHebron: the intervention was implemented in the adult ED in February 2020. Criteria for HCV screening were: age ≥18 years; presenting to the ED; no record of previous serologies; need for phlebotomy for any reason.

SGalicia: regional health service in Galicia, Spain, where the intervention was implemented in 54 primary care sites in the Pontevedra y O Salnés health area in March 2019. Criteria for HIV and HCV screening were: age 18 to 70 years; presenting to primary care; no record of previous serologies; need for phlebotomy for any reason.

SMadeira: regional health service in Madeira, Portugal, where the intervention was implemented in inpatient hospital wards in January 2020, and in the adult ED in July 2020. Criteria for HCV screening were: age 18 to 70 years; in inpatient or seen in the ED; no record of previous serologies; need for phlebotomy for any reason.

GAT: the intervention was implemented in three community-based voluntary counseling and testing (CBVCT) brick-and-mortar sites and 1 mobile unit in March 2019. Criterion for HIV screening was age ≥14, criteria for HBV screening were unvaccinated individual aged ≥14 from a high-prevalence country, and criteria for HCV screening were unvaccinated individual aged ≥14 with a history of exposure to the virus or other known risk factors.

A+: the intervention was implemented in a novel CBVCT site in Torremolinos in April 2019. Criterion for HIV screening was age ≥18, criteria for HBV screening were unvaccinated individual aged ≥18 from a high-prevalence country, and criteria for HCV screening were unvaccinated individual aged ≥18 with a history of exposure to the virus or other known risk factors. Material should be uploaded separately on submission. Please include any supplementary data, figures and/or tables.

### 2.3 Intervention

Each participating HCO appointed:

1. A principal investigator in charge of strategic planning and scientific supervision.
2. A project manager, in charge of implementation and day-to-day management of the intervention.
3. One or more linkage to care navigators, in charge of patient management.

Participating HCOs then implemented the TEST model in 3 phases, with occasional guidance from peer experts from similar organizations.

Screening criteria and workflows were defined, and EHR modifications were used whenever possible to automate eligibility algorithms. Consent procedures were adjusted to ensure opt-out language was used, and refusal to participate in screening was noted in the patient's record. Written consent forms were avoided unless mandated by the local Ethics Committee. Laboratory order forms and patient profiles were updated and EHRs were changed to automatically populate laboratory order forms whenever possible. Biological specimen collection workflows were defined and integrated into standard patient flow. Dedicated testers and rapid tests were limited to CBOs and avoided in formal healthcare settings to facilitate integration and economies of scale. Laboratory testing procedures were updated to ensure reflex testing was used (i.e., positive first-line test results automatically triggered confirmatory testing on the same specimen without the need for physician or patient intervention). Patient notification procedures and linkage to care workflows were defined and assigned to specific people. Each site integrated the redesigned protocols into its policies, trained its staff in the new protocols, and kept an implementation log of monitoring and evaluation indicators that was regularly reviewed to ensure adherence to each TEST pillar. Positive feedback loops were cultivated by sharing key intervention milestones with personnel. Patient education materials and signposting were designed and visibly affixed in public areas.

A detailed description of all implementation activities is included in Supplementary material.

### 2.4 Study of the intervention

A time-series analysis was used to measure the effect of the TEST model over various periods before and after the intervention. Each HCO reported the number of tests performed (HCV RNA tests or/and HIV Ab or HBV Ag) before the intervention and again during and after the intervention. Data was collected in a shared monitoring database provided by laboratory or information technology departments and compiled after being reported by each HCO. Outcomes were analyzed by determining changes in testing volume, as follows:

- Baseline phase: 12-month period prior to the start of the intervention.
- Increase phase: After the introduction of the intervention.
- Disruption phase: During the first wave of the COVID-19 pandemic.
- Sustain phase: After the first wave of the pandemic.

Table 1 shows the specific timeline of each participating center.

**Table 1:** Summary of characteristics of participating HCOs.

**HCO**	**Setting**	**Number of visits/ hospitalizations per year**	**BBVs screened**	**Timeline**		
				**Increase**	**Disruption**	**Sustain**
Hospital de Cascais Dr. José de Almeida (HJA), Portugal	Hospital (ED)	98,000	HIV, HCV	January 19 to February 20 (14 months)	March 20 to May 20 (3 months)	June 20 to December 20 (7 months)
Consorci Hospital General Universitari de València (CHGUV), Spain	Cluster of primary care centers ED, 26 Primary care centers, 3 addiction treatment centers, 1 prison	>3,180,000	HIV, HBV, HCV	February 19 to March 20 (14 months)	April 20 to June 20 (3 months)	July 20 to December 20 (6 months)
Hospital Clínic i Provincial de Barcelona (HCiPB), Spain	Hospital (ED-linked STI clinic)	2,437	HIV, HBV, HCV	October 19 to March 20 (6 months)	April 20 (1 month)	May 20 to December 20 (8 months)
Hospital Universitari Vall d'Hebron (HUVH), Spain	Hospital (ED)	108,000	HCV	February 20 to March 20 (2 months)	April 20 to July 20 (4 months)	August 20 to December 20 (5 months)
Servizo Galego de Saúde (SERGAS), Spain	54 Primary Care sites	224,000	HIV, HCV	April 19 to February 20 (11 months)	March 20 to May 20 (3 months)	June 20 to December 20 (7 months)
Serviço de Saúde da Região Autónoma da Madeira (SESARAM), Portugal	Hospital (wards and ED)	20,000	HCV	January 20 to February 20 (2 months)	March 20 to July 20 (5 months)	Aug 20 to December 20 (5 months)
Grupo de Ativistas em Tratamentos (GAT), Portugal	CBVCT	23,000	HIV, HBV, HCV	March 19 to March 20 (13 months)	April 20 (1 month)	May 20 to December 20 (8 months)
Apoyo Positivo (A+), Spain	CBVCT	600	HIV, HBV, HCV	March 19 to March 20	April 20 (1 month)	May 20 to December 20 (8 months)

CBVCT, community-based voluntary counseling and testing; ED, emergency department; HCO, healthcare organization.

To test whether these changes were likely to be related to implementing the intervention, principal investigators and project managers from participating organizations were asked to participate in structured, open-ended feedback interviews administered by an outside team after the sustain phase. To measure their opinion of the intervention and its effect on transition outcomes, principal investigators and project managers were asked to specify the degree of implementation of each of the 4 pillars of the TEST model and to describe any iterations over PDSA cycles (i.e., Plan, Do, Study, Act). The interviews served to corroborate the statement that the intervention resulted in improved outcomes by examining whether HCOs implemented the intervention framework as intended.

Common qualitative techniques were used to ensure that the principal investigator and project manager interviews were analyzed systematically, including consistent use of the interview guide, audiotaping, and transcription of the interview data.

### 2.5 Measurements

The primary outcome measure was the change in BBV testing rates over time after the intervention (during the increase phase, the disruption phase, and the sustain phase) compared with baseline testing rates (12-month period prior to the start of the intervention) in each HCO. Other variables were also analyzed, including number of patients diagnosed and time from a positive test result to confirmation. Longitudinal data analysis in terms of ANOVA was also carried out to examine the possibility of a significant relation between the changes in the number of tests performed, the type of HCOs and the different study periods.

### 2.6 Analysis

For the analysis of longitudinal data, a mixed ANOVA model was used. The number of tests (dependent variable) in this model is determined by the type of HCO [hospital, primary care center, or community-based voluntary counseling and testing (CBVCT)] and the time period during which the parameters were evaluated (baseline, rise, disruption, or sustain). A possible interaction between the kind of HCO and the time period was considered, and each unique center was entered as a random factor in the model. *P-*values with α = 0.05 were considered to be significant. All analyses were performed in R Core Team (2021) (https://www.R-project.org/) version 4.1.2.

### 2.7 Ethical considerations

Offering BBV screening is considered standard clinical practice in both Spain and Portugal. Local ethics committees were asked to give their opinion when enhanced screening practices required modifying consent procedures from written opt-in consent to oral opt-out consent.

External data management agencies were provided with exclusive monthly aggregate reports on screening production indicators. No patients identifying characteristics were shared outside each HCO.

## 3 Results

Participant HCOs made up a diverse group of organizations, and their implementation of the pillars of the TEST varied accordingly. Table 2 shows a summary of the various levels of TEST implementation as well as testing rate values for each participating institution. T, Testing was successfully integrated into the clinical workflows of all organizations. E, Electronic health record modifications were not implemented in 4 of the 8 participating organizations: A+ is a small CBVCT that did not use an EHR system at the start of the intervention; HValencia opted to include implementation of the SLTC program in their management agreement incentives instead; HValencia and HClinic launched their programs 2 months before the start of the SARS-CoV-2 pandemic, and it was therefore impossible to continue with plans to implement EHR modifications. S, Systemic policy changes were achieved in all organizations, and were approved by all management bodies. T, Training was implemented from the start in all organizations, but repeat training was offered unevenly across HCOs, with decentralized centers facing greater logistic challenges in this regard.

**Table 2:** BBV testing volume variation and adherence to the TEST model in participant organizations.

	**Monthly average persons tested for BBV**							**Use of the TEST Model** ^*^			
	**Baseline (** ***n*** **)**	**Increase (*****n***, +**/–**Δ**%)**		**Disruption (*****n***, +**/–**Δ**%)**		**Sustain (** ***n*** **, %)**		**T Testing integrated in standard care**	**E Electronic health record automations**	**S Systemic policy change adoption**	**T** ***Training and continuous quality improvement***
**Total HCOs**	**3,179**	**6,878**	+**116%**	**2,719**	−**60%**	**5,298**	**77%**				
Formal settings	1,866	5,617	+201%	2,697	−52%	3,961	71%				
Hospitals	240	3,304	+1275%	1,873	−43%	2,650	80%				
HClinic	57	103	+81%	19	−82%	159	154%	A	C	A	B
HCascais	141	1,443	+924%	939	−35%	1,167	81%	A	A	A	A
HVHebron	6	1,426	+23,667%	672	−53%	300	21%	A	C	A	B
SMadeira	36	332	+814%	243	27%	1,024	308%	A	A	A	B
Primary care	1,626	2,313	+42%	824	−64%	1,311	57%				
HValencia	780	920	+18%	69	−92%	214	23%	A	B	A	A
SGalicia	846	1,394	+65%	755	−46%	1,097	79%	A	B	A	B
Community settings	1,313	1,261	−4%	22	−98%	1,337	106%				
A+	27	44	+62%	0	−100%	44	101%	A	C	A	A
GAT	1,286	1,217	−5%	22	−98%	1,293	106%	A	C	A	A

Change in BBV testing rates after the implementation of the intervention was calculated as follow: Increase phase Δ% = (number of tests during the increase phase - number of test at baseline/number of tests at baseline)^*^100; Disruption Δ% = (number of test during the disruption phase - number of test at baseline/number of test at baseline) ^*^100; Sustain phase % = (number of tests during the retain phase/ number of tests during the increase phase). BBV, blood-borne virus; CBO, community-based organization; HCO, healthcare organization. ^*^A = full implementation. B = partial implementation or room for improvement. C = implementation not attempted, not achieved or not possible.

HCOs were successful in scaling up testing, with positive variation from baseline in all but 1 organization (GAT), and a global increase of 116% (ranging from −5% to +23,667%) in testing volume in the “increase” phase, as summarized in Table 1. Prevalence of HIV and HBV was higher in CBOs than in formal healthcare settings (1.2% vs. 0.8% for HIV Ab+; 0.6% vs. 0.5% for HBsAg+). Formal healthcare settings recorded the largest increases in testing volume (201%); of these, primary care recorded a more modest increase (42%), while hospital-based models of care recorded a global 14-fold (1,275%) increase in testing. HValencia was unsuccessful in implementing the screening in its ED due to the imposition of written opt-in consent forms by its local Ethics Committee, which proved too burdensome for the fast-paced environment of a large ED.

The first period of COVID-19 pandemic-related restrictions on freedom of movement (variously described as stay-at-home orders, shutdowns, or lockdowns) lasted from 15 March to 25 April 2020 in Spain, and from 19 March to 4 May 2020 in Portugal. All organizations observed an immediate combined 60% decrease in testing volume in the “disruption” phase. CBVCTs were the most affected sites, with a global 98% decrease in testing volume as lockdowns forced them to halt all activities. Formal healthcare settings observed a global 52% decrease in testing volume with heterogeneous effects depending on specific settings and the degree of implementation of the TEST model. HCOs that implemented SLTC protocols targeting patients seeking secondary care observed the smallest reduction in testing volume, at 43%. In contrast, those targeting patients in a primary care setting observed a larger reduction of 64%. The HCOs were most successful in implementing the “E” pillar of the TEST model — meaning using their EHR systems to automate patient eligibility assessment and laboratory orders— observed the smallest reduction in testing volume, at 31% (HCascais, SMadeira). In comparison, HCOs that did not use their EHR systems for this purpose observed a larger reduction of 78% (HVHebron, HClinic, A+).

**Figure 1:**
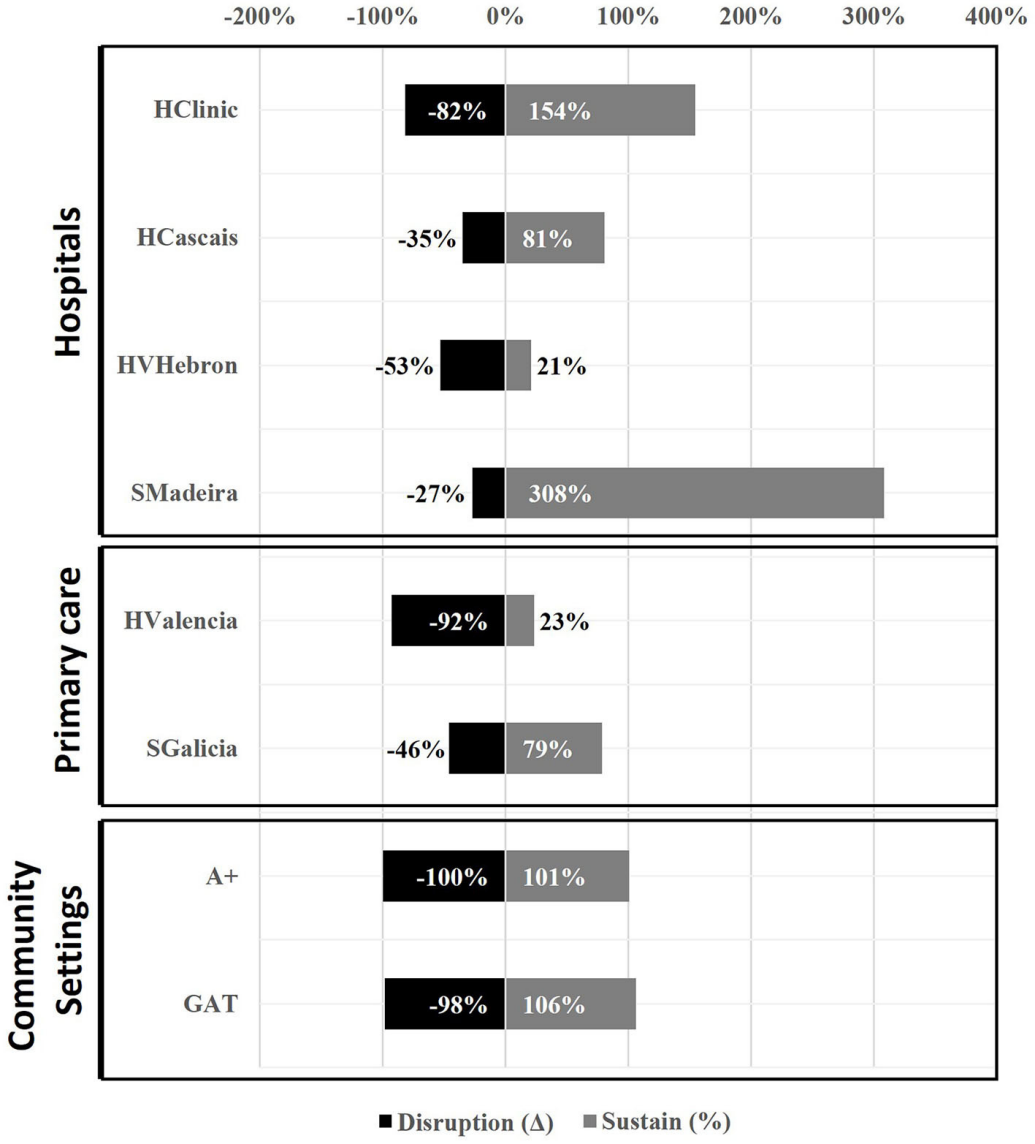
Blood borne viruses testing volume variation at participant organizations during the “disruption” phase and proportion of recovery in the “sustain” phase.

As COVID-19 lockdowns were lifted, organizations recovered 77% of their average monthly testing in the “sustain” phase. As shown in Figure 1, CBVCTs showed the fastest, fullest recovery of testing volume post reopening, recovering 106% of their average monthly testing within 2 months. Although they were more vulnerable to the effects of restrictive pandemic control measures, they also showed the highest resilience of all organizations analyzed, further strengthening the case for investing in community-based organizations involved in BBV prevention. Formal healthcare settings, globally, had recovered 71% of their average monthly testing within 4 months. HCOs showed the strongest recovery in the “E” pillar of the TEST model, with a global 123% recovery of average monthly testing vs. 46% recovery in those not using EHR automations.

Although HVHebron initially showed one of the most remarkable increases in testing, it only recovered 21% of its average monthly testing volume after the lockdowns – the lowest of all HCOs. Following feedback from frontline ED staff and implementation of PDSA cycles, HVHebron has since changed its protocol and reduced the extent of human intervention in their enhanced SLTC program, thereby implementing the tenets of the “E” pillar of the TEST model. As of December 2021, HVHebron had managed to increase its testing output to 56% of its average monthly testing from before lockdowns.

The ANOVA test showed differences between the time periods evaluated, although they were not statistically significant (*p* = 0.059) (Table 3). There were no statistical differences between types of HCO (*p* = 0.774) when adjusting for time trends, neither in the interaction between different time trends and types of HCO (*p* = 0.296).

**Table 3:** Summary of the ANOVA test.

	**F (DFn, DFd)**	***p*-value**
Health care organizations	0.38 (2, 5)	0.774
Time periods	3.09 (3, 15)	0.059
Health care organizations: time	1.35 (6, 15)	0.296

DF, degrees of freedom.

In addition to testing scale-up, other aspects were considered when assessing the merits of this intervention. The implementation of enhanced screening protocols allowed HCOs to diagnose a total of 1,860 patients over the study period (i.e., 1,139 HIV Ab+, 285 HBsAg+, 436 HCV RNA+, Table 4), of which 1179 were diagnosed during the “increase” phase, 79 during the “disruption” phase and 607 during the sustain phase. It is unlikely that these 79 patients (i.e., 58 HIV Ab+, 3 HBsAg+, 18 HCV RNA+) would have been diagnosed during this lockdown period were it not for the enhanced SLTC protocols, considering the lockdowns and travel restrictions. HCOs also diagnosed 4 acute HIV infections (AHI) over the study period. Since patients presenting with AHI have the highest likelihood of transmission to others, this is a significant contribution to individual and public health. HValencia recorded remarkable improvements in diagnosing late HIV presentation, from 52% to 33%, and diagnosed 94% and 69% of HBV and HCV patients, respectively, before the onset of severe liver disease. HCascais recorded an even more remarkable decrease in late HIV presentation, from 78% to 40% of patients diagnosed in the ED.

**Table 4:** Blood borne viruses diagnoses.

	**HIV+ (Ab+)**	**HBV+ (Ag+)**	**HCV+ (RNA+)**	**BBV+**
Total HCOs	1,139	285	436	1,860
Formal settings	780	102	366	1,248
Hospitals	738	14	202	954
HClinic	496	14	116	626
HCascais	242	-	26	268
HVHebron	-	-	44	44
SMadeira	-	-	16	16
Primary care	42	88	164	294
HValencia	23	88	71	182
SGalicia	19	-	93	112
Community settings	359	183	70	612
A+	24	2	4	30
GAT	335	181	66	582

BBV, blood-borne virus; CBO, community-based organization; HCO, healthcare organization.

## 4 Discussion

Testing volumes increased by 116% after the launch of the TEST model for enhanced SLTC, with formal healthcare settings recording larger increases than CBOs. While 1 participating CBO (A+) recorded a 62% increase in testing in the increase phase, some other (GAT) experienced a 5% decrease in testing due to staff budget control measures that reduced patient consultation hours over that period. Although our primary outcome measure of success was changes in testing volume, other variables were also meaningfully impacted. Specifically, the number of BBVs diagnoses were also enhanced (1860 diagnoses over the study period) and time from a positive test result to confirmation was reduced from weeks to hours following implementation of point of care RNA reflex testing in GAT. Although this significantly improves SLTC practices, it was not reflected in an increase in testing.

The effect of the implementation of the screening intervention and the impact of the COVID-19 pandemic differed according to the type of HCO analyzed. In CBVCTs, BBV testing was practically brought to a standstill during lockdowns. However, once they opened after the worst phase of the pandemic, tests numbers rapidly increased to pre-lockdown rates. One of the reasons for the fast recovery in CBVCTs was the high demand for pre-exposure prophylaxis (PrEP) after reopening, showing that levels of sexual activity did not vary during lockdowns. In addition, the 2 participating CBVCTs quickly adapted to the epidemiologic situation by implementing measures to increase their testing capacity, such as increasing the number of patient examination rooms and opening tents next to mobile units. These strategies, together with an increase in social media activity, led to a rapid recovery in testing rates. In contrast, formal settings in which the intervention was implemented in the ED (HCascais, HClinic), lockdowns did not drastically reduce testing compared to pre-pandemic rates, particularly if automation had been implemented, as EDs remained open. Furthermore, individuals seeking care for COVID-19 might have been tested for HIV, since the same social determinants of health increase the risk of infection in both diseases (30). In addition, individuals with symptomatic but untreated HIV might also have been at increased risk of symptoms requiring emergency care. Indeed, during the pandemic, patients with HIV have been shown to be more likely to seek emergency care than primary care or telehealth services (31).

Similar results have been reported in other countries. For instance, in an urban ED of a US hospital with a universal HIV screening program also including automated EMR, the volume of testing performed during the pre-pandemic period and pandemic period was not significantly different, in agreement with what we found in the formal settings in which testing intervention was implemented in the ED (32). On the other hand, a European survey on 71 CBVCTs services from 28 countries checking on the impact of COVID-19 pandemic on BBVs testing demonstrated a very major decrease (>50%) in the volume of testing for all the infections in this setting, in agreement with the results of our study (33). However, it is important to point out that while BBV screening should be broadly encouraged, it should also be tailored to the various situations. The opportunistic opt-out screening approach used in this study helps minimize any factors that might discourage participation, ensuring that individuals have the freedom to decline this or any other routine clinical investigations, thereby respecting their right to make informed choices about their healthcare (29).

However, in some cases, particularly in locations where disease prevalence is low, targeted screening to high-risk population may be more effective and cost-efficient. In those cases, it is important to establish appropriate default risk criteria to guide decision-making. Key considerations for defining default risk criteria include gather and analyse accurate and up-to-date prevalence data specific to the region or setting in question, consider the local epidemiological factors, including the incidence and distribution of BBVs, conduct a comprehensive risk assessment, refer to relevant national and international guidelines and regulations among others.

Although the COVID-19 pandemic reduced the number of in-person visits, it drove the development of other health care services, including telehealth, which are often used to educate patients and increase awareness of the importance of preventive care. However, despite efforts to provide alternatives to in-person care, COVID-19 and the resulting disruption of health systems may increase new infections and mortality rates for years to come.

Interestingly, some institutions reported that the profile of patients accessing testing during the pandemic differed with respect to the pre-pandemic period. Both SGalicia and SMadeira observed an increase in the proportion of non-native patients accessing care. Due to travel and work restrictions during the pandemic, immigrants might have remained in those communities longer than planned, forcing them to seek health care in their host countries. A considerable proportion of patients seen in HVHebron during lockdowns were also vulnerable immigrants. According to this hospital in Barcelona, patients with psychiatric and psychological disorders were also more frequently seen. This may correlate with a higher prevalence of psychiatric disease among people living with HIV or HCV (34, 35) for whom mental health care services may have been disrupted (36), while COVID-19 has exacerbated mental health conditions such as depression, social isolation, emotional distress, and substance abuse both in the general population and in people living with HIV (37, 38).

Our findings highlight the importance of using EHR systems to automate screening. HCOs that used their EHR systems to automate patient eligibility assessment and laboratory orders (fully implementing the tenets of the “E” pillar of the TEST model, level A in Table 1) had the smallest reduction in testing volume compared with organizations not using their systems for this purpose (partial or no implementation of the “E” pillar, levels B and C in Table 1) with 31% vs. 78%, respectively. Integration of HIV, HBV, and HCV into the flow of other laboratory tests already performed by HCOs through automation of the EHR and other technologies enables some patients to receive their serological results before discharge, thus facilitating timely disclosure, counseling, and referral, and avoiding the need for a follow-up appointment to give them their positive result and refer them to outpatient care, which is frequently challenging. However, implementation of automations in some organizations is both technically difficult and time consuming.

Participation in the intervention resulted in a positive culture change among healthcare professionals and communities alike, and both groups stated, subjectively, that awareness of the importance of screening for BBVs has increased. Patient attitudes toward enhanced screening practices were determined by recording the rate of refusal to undergo screening, which the 2 HCOs that specifically recorded refusal rates among patients eligible for screening (HCascais and SMadeira) estimated at <10%. This shows that the intervention was successful in reducing the stigma associated with testing.

Our study has several limitations. We did not conduct a systematic audit of culture and practices, and thus our assessments may contain some inaccuracies. We were unable to adjust for unmeasured confounders and did not evaluate possible modifiers of the effect of factors such as HCO size, number of healthcare workers involved, and other environmental factors. Existing SLTC practices differed considerably among participating HCOs, as did eligibility criteria for screening (e.g., different age cut-off points for inclusion/exclusion in screening, or symptoms suggestive of infection, in 1 case). The exact duration, in months, of each phase of implementation (i.e., baseline, increase, disruption, sustain) also differed among HCOs, as they were included in the project on a rolling basis and lockdown restrictions varied according to geography. Those differences in the times or seasons where the intervention was implemented could potentially expose individuals to varying environmental factors or disease prevalence and impact the observed results. Also, the heterogeneity in baseline risk (ED patients may inherently present with higher baseline risks, whereas primary care or organization-based screenings may target populations with different risk profiles) can influence screening outcomes.

The data presented correspond to entries made by participants in a shared monitoring database provided by laboratory or information technology departments. The figures could not be externally verified due to information governance issues and are thus susceptible to error. Our study compared performance with historical baseline testing volume rates. The quality of these data was called into question in some HCOs, particularly regarding second-line confirmatory testing. In addition to confirming new infections, HIV and HCV RNA tests may also be requested to monitor known infections, and this may have given a skewed perception that baseline practices were higher than they really were when appropriate control measures were not put in place (e.g., coding tests according to patient profile or requesting department). These potential errors were eliminated by using first-line antibody and serology tests alone as our baseline, as these are not repeated for patients already in care.

The degree of implementation of the TEST model is likely highly context-dependent, and limits the extent to which our results can be extrapolated to other organizations. Some HCOs did not have the resources or autonomy to fully implement the intervention. Some formal healthcare organizations in Spain and Portugal may lack the quality improvement culture needed to consistently support an iterative process that requires changes to be made at various time points, other than merely at the start of the intervention. This was somewhat mitigated by comparing our results with other interventions implementing the TEST model in the US (23–27) and conceptually similar interventions in the UK, which reported a 78% increase in HIV testing (39).

In interpreting the effect of the TEST model, we may have overlooked some positive and negative outcomes, such as the possible halo effect caused by increased healthcare staff awareness of the importance of screening, which could have increased testing beyond the established protocol, as seen in primary care centers in the HValencia. On the other hand, because staffs were aware that patients would likely be enrolled in screening where EHR modifications had been made, they could have reduced screening for indicator conditions and situations associated with high BBV prevalence, as seen in the ED of HCascais. This could be corrected by staff re-training or by including indicator conditions as further triggers for screening.

Although participant organizations also monitored their healthcare navigation process and corresponding LTC rates to ensure patients attended a first post-diagnosis visit with a specialist, we did not analyse this information in this study due to data quality concerns. Some HCOs refer patients to other organizations for care, and these do not always provide timely or accurate feedback on the success of patient LTC. Other organizations refer patients to in-house departments but record LTC rates of 100% which, according to the literature, are likely to be inaccurate. Where data quality was consistent, average LTC rates increased at the start of the intervention, only to decrease with the introduction of lockdown. However, further research is needed to establish the impact of the intervention and of the pandemic on LTC, an integral part of any meaningful screening program.

Our study did not include factors relating to cost-effectiveness analysis (CEA) of this quality improvement intervention, which is crucial for policymaking at the regional or national scale. However, previous CEA have found that HIV screening in the population is cost-effective for antibody prevalence of ≥0.1% in the US (40), ≥0.2% in the UK (41), and ≥0.06% in Portugal (42). On the other hand, HBV screening in the general population is cost-effective for HBsAg prevalence of ≥0.3% in the US (43), and ≥0.25% in the UK (41). For HCV screening in the general population, it is cost-effective for antibody prevalence of ≥0.07% in the US, HCV RNA prevalence of ≥0.26% in the UK (41) ≥0.13% in Spain (44) and ≥0.16% in Italy (45). Since we estimate that the cost of consumables and human resources is lower in Spain and Portugal than in the UK and the US, and that the HIV, HBV, and HCV prevalence figures found in our study are equal to or higher than the afore-mentioned thresholds, we hypothesize that BBV screening in the study populations will also be cost-effective in Spain and Portugal. However, this needs to be confirmed in detailed CEA studies.

## 5 Conclusions

Implementation of the TEST model enabled HCOs to increase and sustain BBV screening, even during COVID-19 lockdowns. Considering the ECDC's call for countries to increase test coverage and uptake, and the worldwide disruptions in BBV screening following the start of the SARS-CoV-2 pandemic, our study shows that effective, resilient, evidence-based models can increase screening. Additional research is needed to develop equally resilient patient linkage to care models in challenging times, and to assess the cost-effectiveness of this strategy.

## Data availability statement

The raw data supporting the conclusions of this article will be made available by the authors upon reasonable request.

## Ethics statement

Gilead Sciences' FOCUS Program funding supported screening and linkage to a first healthcare appointment after diagnosis, regardless of subsequent patient management. The studies involving humans were approved by the Local Ethics Committees of the eight participating centers. The studies were conducted in accordance with the local legislation and institutional requirements. The Ethics Committee/institutional review board waived the requirement of written informed consent for participation from the participants or the participants' legal guardians/next of kin because Offering BBV screening is considered standard clinical practice in both Spain and Portugal. Local Ethics Committees were asked to give their opinion when enhanced screening practices required modifying consent procedures from written opt-in consent to oral opt-out consent.

## Author contributions

IV-P: Conceptualization, Formal analysis, Methodology, Supervision, Validation, Writing—original draft, Writing—review & editing, Investigation. EO: Methodology, Supervision, Validation, Writing—review & editing, Conceptualization, Investigation. IC: Methodology, Supervision, Validation, Writing—review & editing, Conceptualization, Investigation. MB: Methodology, Supervision, Validation, Writing—review & editing, Conceptualization, Investigation. JT-V: Methodology, Supervision, Validation, Writing—review & editing, Conceptualization, Investigation. VM-P: Methodology, Supervision, Validation, Writing—review & editing, Conceptualization, Investigation. MR: Methodology, Supervision, Validation, Writing—review & editing, Conceptualization, Investigation, Project administration, Resources. JG: Methodology, Supervision, Validation, Writing—review & editing, Conceptualization, Investigation. CE-S: Project administration, Writing—review & editing, Data curation, Investigation. MG: Project administration, Writing—review & editing, Data curation, Investigation. TM: Project administration, Writing—review & editing, Data curation, Software. MM: Project administration, Writing—review & editing, Data curation. VG: Project administration, Writing—review & editing, Data curation. JL-A: Project administration, Validation, Writing—review & editing, Data curation, Investigation, Methodology, Software, Supervision. AB-D: Project administration, Writing—review & editing, Data curation. SP: Project administration, Writing—review & editing, Data curation, Software. SD: Project administration, Writing—review & editing, Data curation. EX: Project administration, Validation, Writing—review & editing, Data curation, Investigation. MV: Project administration, Writing—review & editing, Data curation, Resources, Supervision. GG: Project administration, Writing—review & editing, Data curation, Investigation, Supervision. MH: Project administration, Writing—review & editing, Data curation. DM: Conceptualization, Data curation, Formal analysis, Supervision, Validation, Writing—review & editing, Funding acquisition, Investigation, Methodology, Project administration, Resources, Writing—original draft. MG: Methodology, Supervision, Validation, Writing—review & editing, Conceptualization, Investigation.
