# Supplementary material for: Increasing and sustaining blood-borne virus screening in Spain and Portugal throughout the COVID-19 pandemic: a multi-center quality improvement intervention
Source: Front Public Health. 2024 Jan 24;11:1268888. doi: 10.3389/fpubh.2023.1268888 (PMC10847218; doi:10.3389/fpubh.2023.1268888)
Supplement: Supplementary file 1 [file Table_1.DOCX]

Supplementary Material

Increasing and sustaining blood-borne virus screening in Spain and Portugal throughout the COVID-19 pandemic: a multi-centre quality improvement intervention

**Inês Vaz-Pinto MD MSc,^1*^ Enrique Ortega MD MPH MHA PhD,^2^ Ivan Chivite MD,3 María Butí MD PhD,^4,5^ Juan Turnes-Vázquez MD MSc PhD,^6^ Vítor Magno-Pereira MD,^7^ Miguel Rocha RN MSN,^8^ Jorge Garrido lic. OT,^9^ Catarina Esteves RN,^1^ Mafalda Guimaraes MD,^1^ Tomás Mourão BA,^1^ María Martínez Roma PharmD MSc,^2^ Vanessa Guilera RN,^3^ Jordi Llaneras-Artigues MD MSc PhD,^4^ Ana Barreira-Díaz MD,^4^ Santiago Pérez Cachafeiro MD MSc,^6^ Sandra Daponte Angueira,^6^ Elisa Xavier RN MSc,^7^ Marianna Vicente PSY,^8^ Gema Garrido MSW,^9^ Maria Teresa Heredia MSCP,^9^ Diogo Medina MD MPH MBA^10^, Miguel García Deltoro MD PhD.^2^**

*** Correspondence:** Corresponding Author: email@uni.edu

# Supplementary Data

**TEST model implementation checklist**

**Planning**

- Organization assigns **people** to oversee and manage the project and shows engagement of internal and external stakeholders in the project planning stage (e.g., management, heads of departments, laboratory personnel, IT personnel, external service providers, health authorities, patient associations).
- Organization demonstrates **knowledge** of local epidemiology and baseline metrics, as evidenced by incidence or prevalence of BBV infection, proportion of individuals unaware of their status, number of patients in care per year, new patients entering care if applicable, age distribution, patients requiring phlebotomy, patients undergoing BBV serology, reactive confirmed cases, patients linked to care, and linkage to care pathway wait times.
- Organization states that **screening strategies** are either provider-initiated or systematic, targeted or universal, and lists eligibility criteria. Coexisting strategies for different viruses or settings are acknowledged (e.g., systematic universal screening for HIV in EDs and provider-initiated targeted screening in outreach settings for HCV).
- Organization states that **screening frequency** is either lifetime (e.g., more common for HBV and HCV screening in the general population), annual (e.g., more common for HIV screening) or other (e.g., reported or recorded risk factors may prompt more frequent screening).
- SLTC project includes a clear **before/after scenario** based on stated assumptions, timeline, budget, and continuous QI plan based on stakeholder input, staff training, data audit and analysis.

**Screening**

- The screening **eligibility** workflow is defined and EHR systems, if in place, are changed to automate eligibility determination algorithms,. On-screen prompts are avoided unless mandatory or necessary.
- **Consent procedures** are adjusted to ensure opt-out language is used (e.g., staff inform patient that “It is our organization’s policy to offer testing to everyone. We’ll test you unless you say no.”), and consent forms are updated to include BBV testing as a standard of care, if generalized consent forms are in use. Separate written consent forms are avoided unless mandatory.
- **Laboratory order** forms are updated and EHRs are changed to automatically populate order forms.
- **Biological specimen collection** workflow is defined and integrated into standard patient flow. Dedicated testers and rapid tests are avoided in formal health care settings unless necessary (e.g., outreach settings).
- Laboratory testing procedures are updated to ensure **reflex testing** is implemented (i.e., positive first-line test results automatically trigger confirmatory testing on the same specimen without the need for physician or patient intervention).

**Linkage to Care (LTC)**

- **Patient notification** procedures are defined, protecting patient confidentiality by prohibiting communication through relatives, voicemail, corporate email addresses or detailed exchanges. If needed, envelopes omit hospital department names and letters are deliberately vague in specifics (e.g., “Further investigations are needed to address recent test results obtained at our institution. Please call us to make an appointment at your earliest convenience using the number provided in this letter ”).
- **LTC workflow** is defined and assigned to specific people in the organization (i.e., LTC officers or navigators, ideally social workers, psychologists, therapists, nurses, or trained peers), who receive alerts of reactive and confirmed test results at defined intervals (e.g., weekly, daily, or in real time), and secure specialist appointments in a timely manner (i.e., ideally within a maximum of 7 days for HIV and 30 days for viral hepatitis).
- LTC activities are supported by adequate **patient tracking tools** to facilitate identification, linkage, tracking, and confirmation of care. Examples range from spreadsheets to customer relationship management software.
- LTC officers work to overcome patient unwillingness to receive care, making **appropriate concessions** (e.g., allowing up to 3 missed phone calls or visits before considering a patient not linked to care) and ensuring staff is alerted should a known positive patient who has not yet been linked to care be readmitted

**Continuous Quality Improvement**

- **Patients are informed** of the organization’s new BBV SLTC policy via visible signposting and/or multimedia.
- **Staff is informed** of the organization’s new BBV SLTC policy via direct in-person and written communication by management and/or the clinical director (e.g., face-to-face project pre-launch session, mailings), and receives **initial and refresher training** on the adapted workflow, laboratory forms, EHR, and opt-out language.
- Project **data is extracted** at least monthly and automatically by the EHR system, rather than manually by a data manager. IT changes are used to automatically export project variables.
- Project data analysis assesses and acts on **screening penetration rate** (i.e., patients eligible for screening being tested), by generating positive feedback reports on adherence by site or provider and reaching out to underperformers (e.g., "We understand you are having a hard time enrolling patients in screening and would like to hear how we can help make it easier for you to do so.")
- Results of SLTC project are **shared with the staff** at regular intervals (e.g., quarterly), with express recognition of their contribution.
- There are specific plans to **disseminate the results of the project** through publications in peer review journals and national or international conferences, as well as with relevant health authorities and policy makers.
